# Effects of a Type I RM System on Gene Expression and Glycogen Catabolism in *Synechocystis* sp. PCC 6803

**DOI:** 10.3389/fmicb.2020.01258

**Published:** 2020-06-09

**Authors:** Dongqing Wu, Yali Wang, Xudong Xu

**Affiliations:** ^1^State Key Laboratory of Freshwater Ecology and Biotechnology, Institute of Hydrobiology, Chinese Academy of Sciences, Wuhan, China; ^2^Key Laboratory of Algal Biology, Institute of Hydrobiology, Chinese Academy of Sciences, Wuhan, China; ^3^University of the Chinese Academy of Sciences, Beijing, China

**Keywords:** *Synechocystis*, type I RM system, gene regulation, *glgP*, glycogen catabolism

## Abstract

Increasing evidence has shown that DNA methylation is involved in gene regulation in prokaryotes. However, there have been very limited reports about the role of DNA methylation in regulation of gene expression and physiological functions in cyanobacteria. In *Synechocystis* sp. PCC 6803, four genes on the plasmid pSYSX are predicted to encode the type I restriction-methylation system, *slr6095* and *slr6096* for the M subunit, *slr6097* for the S subunit and *slr6102* for the *R* subunit. Compared to the wild type, *slr6095*, *slr6096*, and *slr6097* mutants lacked the GG^m6^AN_7_TTGG/CCA^m6^AN_7_TCC methylation in genomic DNA. Transcriptomic analysis indicated that 171 genes were reproducibly up- or down-regulated in all three mutants relative to the wild type. The changed expression of some genes, including *sll1356* for glycogen phosphorylase (GlgP), was associated with the loss of GG^m6^AN_7_TTGG/CCA^m6^AN_7_TCC methylation in the coding regions or the upstream non-coding sequences. Inactivation of *slr6095*, *slr6096*, or *slr6097* increased the expression of *sll1356* and the GlgP activity but lowered the glycogen content. These results indicated that the DNA methylation by a type I RM system could alter the expression of certain genes and physiological functions in *Synechocystis* sp. PCC 6803.

## Introduction

DNA methylation as an epigenetic signal is found in both eukaryotes and prokaryotes. In bacteria, a methyl group from S-adenosyl-L-methionine (AdoMet) is transferred to cytosine or adenine, resulting in *N*^4^-methyl-cytosine, *C*^5^-methyl-cytosine, or *N*^6^-methyl-adenine on DNA (Sánchez-Romero and Casadesús, 2020). Because methyl groups located in the major groove of double helix alter protein-DNA interactions and DNA curvature, DNA methylation may protect DNA from cleavage by restriction enzymes and regulate gene expression, cell cycle, chromosome replication, phase variation, virulence, and mismatch repair (Sánchez-Romero and Casadesús, 2020). DNA methylation is catalyzed by DNA methyltransferases (MTases), which include those of restriction-modification (RM) systems and orphan methylases (such as Dam and Dcm in *Escherichia coli*; Blow et al., 2016).

Cyanobacteria are a group of prokaryotes that perform oxygenic photosynthesis (Castenholz, 2001). Restriction-methylation systems have been predicted in genomes of most cyanobacteria. In addition, some orphan methylases were identified. In *Anabaena* sp. PCC 7120, a heterocyst-forming filamentous cyanobacterium, there are four predicted orphan MTases, denoted as Dmt A to D, in addition to the MTases of RM systems (Matveyev et al., 2001). These orphan MTases modifiy DNA at GATC (DmtA), GGCC (DmtB), CGATCG (DmtC), or RCCGGY (DmtD). In *Synechocystis* sp. PCC 6803 (hereafter *Synechocystis* 6803), a unicellular cyanobacterium, there are at least five different methylation activities toward specific DNA sequences: M.Ssp6803I for ^m5^CGATCG (Scharnagl et al., 1998), M.Ssp6803II for GG^m4^CC, M.Ssp6803III for G^m6^ATC, M.Ssp6803IV for GA^m6^AGGC, and M.Ssp6803V for the bipartite motif GG^m6^AN7TTGG/CCA^m6^AN7TCC (Hagemann et al., 2018). DNA methylation may play roles in cellular and physiological functions. For an example, the GG^m4^CC methylation is involved in regulation of gene expression, fine-tuning of DNA replication and DNA repair in *Synechocystis* sp. PCC 6803 (Gärtner et al., 2019).

In cyanobacteria, glycogen is the most important storage polysaccharide. It is an α-1,4 linked α-1,6 branched glucose polymer. The synthesis of glycogen depends on ADP glucose pyrophosphorylase, glycogen synthase, and a branching enzyme (Preiss, 1984), whereas the degradation of glycogen depends on glycogen phosphorylase (GlgP), the debranching enzyme, α-1,4-glucanotransferase, and maltodextrin phosphorylase (Ball and Morell, 2003). GlgP is the key enzyme involved in glycogen digestion. In *Synechocystis* 6803, there are two GlgP genes, *glgP1* (*sll1356*), and *glgP2* (*slr1367*), which are functionally diverged (Fu and Xu, 2006) and differently regulated (Osanai et al., 2014). Under autotrophic growth conditions, *glgP1* plays a more important role in glycogen digestion (Fu and Xu, 2006).

When we analyzed the previously reported DNA methylase M.Ssp6803V (*slr6095*), we realized that it is probably not an orphan methylase as proposed, because *slr6095* (N-terminal portion of M subunit, for methylation), *slr6096* (C-terminal portion of M subunit), *slr6097* (S subunit, for recognition specificity), and *slr6102* (R subunit, for restriction) are predicted to encode a type I RM system. In this type of RM system, M and S subunits are required for the restriction activity as well (Murray, 2000), therefore their encoding genes can be inactivated in the presence of a functional R subunit gene. We generated mutants for *slr6095*, *slr6096*, and *slr6097*, and analyzed differences in genome-wide gene expression and DNA methylation between mutants and the wild type strain. Inactivation of these genes consistently abolished DNA methylation at GG^m6^AN_7_TTGG/CCA^m6^AN_7_TCC motifs in the genome and indirectly altered the expression of many genes and the glycogen catabolism in *Synechocystis* 6803.

## Materials and Methods

### Strains and Culture Conditions

*Synechocystis* sp. PCC 6803 and derivatives were cultured in BG11 in flasks on a rotary shaker at 30°C under continuous illumination of 30 μE m^–2^ s^–1^. Kanamycin (25 μg mL^–1^) was added as needed.

### DNA Preparation

Genomic DNA was extracted using the cetyltrimethylammonium bromide (CTAB) method (Murray and Thompson, 1980) with modifications. The cyanobacterial cells were broken by grinding in liquid nitrogen and suspended in CTAB solution. The broken cell suspension was treated with proteinase K (100 μg mL^–1^) and β-mercaptoethanol (2%, v/v) at 65°C for 1 h and centrifuged at 17,226 *g* for 30 min. The supernatant was extracted with phenol:chloroform:isoamyl alcohol (25:24:1), followed by chloroform:isoamyl alcohol (24:1). Nucleic acids were precipitated with isopropanol, washed with 70% ethanol, dissolved in TE buffer. After removal of RNA with RNase A (100 μg mL^–1^), DNA was extracted with phenol and chloroform, precipitated with ethanol and dissolved in TE buffer. The quality of DNA was assessed by agarose-gel electrophoresis and OD_260_/OD_280_ (1.8∼2.0).

### RNA Preparation

Total RNA was extracted using Trizol reagent (Invitrogen, Carlsbad, CA, United States) according to the manufacturer’s instructions. After removal of DNA with DNase RQ1 (Promega, Madison, WI, United States), the sample was extracted with Trizol reagent again. RNA was precipitated with isopropanol, washed with 70% ethanol and dissolved in nuclease-free ddH_2_O. RNA integrity was verified on Bioanalyzer (Agilent, Santa Clara, CA, United States) using RNA 6000 nano chip, and the RNA integrity numbers (RIN) were shown to be >6.5.

### Construction of Plasmids and Generation of Mutants

Molecular manipulations were performed according to manufacturers’ instructions. Clones of PCR products were confirmed by sequencing. Construction of plasmids and primers used are described in Supplementary Table S1 in the Supplementary Material.

Using overlap PCR, an *Xho*I site was introduced into *slr6095*, *slr6096*, and *slr6097*, and the DNA fragment containing one of these genes, interrupted by the *Xho*I site, was cloned into pMD18-T, then a kanamycin-resistance (Km^r^) cassette was inserted into the *Xho*I site, producing plasmids pHB6515, pHB6520, and pHB6517. These plasmids were used to inactivate *slr6095*, *slr6096*, and *slr6097* in *Synechocystis* 6803.

Transformation of *Synechocystis* 6803 was performed as described by Williams (1988). Briefly, 100 μl of cell suspension was mixed with DNA (10 μg mL^–1^) and incubated for 4 to 6 h at 30°C in the light of 30 μE m^–2^ s^–1^, spread onto membrane filters resting on BG11 agar plates and allowed to grow for 20 h. The filters were then transferred to plates supplemented with glucose (5 mM) and kanamycin (25 μg mL^–1^). Transformants usually appeared within 4 days. After streaking on plates, the transformants were cultured in liquid BG11 with kanamycin. The complete segregation of mutants was confirmed by PCR. *Synechocystis* strains and primers used are described in Supplementary Table S1 in the Supplementary Material.

### SMRT (Single Molecule Real Time) Sequencing and DNA Methylation Analysis

DNA methylation was analyzed by whole-genome SMRT sequencing. Briefly, libraries with 10 kb inserts were prepared according to the PacBio Sequel Microbiological sequencing protocol, and the genome DNA was sequenced with a PacBio Sequel platform. DNA methylation was then analyzed with the SMRT raw data using the SMRT LINK 6.0.0 software^1^. Base modifications and motifs were analyzed using P_ModificationDetection and P_MotifFinder module from the SMRT Analysis software. Because SMRT sequencing is not suitable for ^m5^C methylation (Murray et al., 2012), we collected the data for ^m6^A and ^m4^C only (consistent between two biological repeats). Raw sequence read data has been deposited in the NCBI Sequence Read Archive (SRA) with the study identifier SRP256040.

### RNA Sequencing and Differential Expression Analysis

*Synechocystis* 6803 and mutants were cultured in BG11 to an OD_730_ of 1.0 at 30°C in the light of 30 μE m^–2^ s^–1^. Total RNA was extracted using TRIzol reagent (Invitrogen, Carlsbad, CA, United States). DNA was removed with RNase-free DNase I (Promega, Madison, WI, United States). RNA was precipitated with isopropanol and stored in 70% ethanol. Strand-specific RNA-seq libraries were prepared using the Illumina small RNA sample preparation kit. RNA sequencing was carried out using the HiSeq 2500 sequencing instrument (Illumina, San Diego, CA, United States) to generate paired-end reads with a length of 150 bp. Reads were aligned against the Cyanobase^2^ genome sequence for *Synechocystis* 6803 using TopHat (Trapnell et al., 2009). Differential gene expression analysis was performed by using the DESeq package (Anders and Huber, 2010). Fold changes of >2 or <0.5 with *P*-value of <0.01 were defined as differentially expressed. Data are means ± SD produced from 3 biological replicates. Raw sequence read data has been deposited in the NCBI SRA with the study identifier SRP258660.

### Reverse-Transcriptase Quantitative Polymerase Chain Reaction (RT-qPCR)

Reverse transcription and the RT-qPCR analysis were performed according to Gao and Xu (2012). *rnp*B (RNase P subunit B; Vioque, 1992) was used as the internal control. PCR primers (indicated with “RT” in name) are listed in Supplementary Table S1. Data are means ± SD produced from 3 technical repeats.

### Determination of Glycogen Contents and Glycogen Phosphorylase Activity

*Synechocystis* 6803 and mutants were cultured in BG11 to an OD_730_ of 1.0. Glycogen contents and GlgP activities were determined according to previously described (Fu and Xu, 2006). Data are means ± SD produced from 3 biological replicates.

### Prediction of Gene Functions

The type I RM system was predicted according to the REBASE^3^ and the Cyanobase^4^. Structural domains were predicted using SMART analysis^5^. Similarity searches were performed using Blastp^6^. Multiple sequence alignment was performed using the ClustalW^7^.

## Results

### Gene Cluster Analysis and Mutant Construction

**FIGURE 1:**
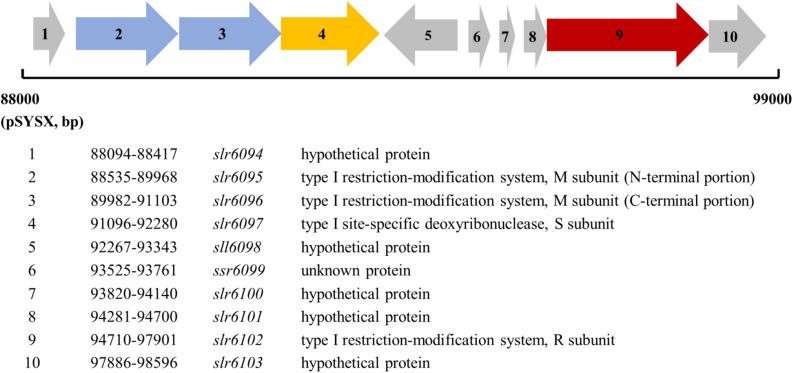
Genes encoding the type I restriction-modification system on the plasmid pSYSX in *Synechcystis* 6803.

*slr6095* is located in a gene cluster on the plasmid pSYSX (Figure 1). According to the REBASE and the Cyanobase, *slr6095*, and *slr6096* are predicted to encode two portions of the M subunit of a type I RM system, *slr6097* encodes the S subunit, *slr6102* the R subunit. Alignment with N6-Mtases from *Rhizobiales bacterium* and *Thiorhodovibrio* sp. 970 shows that Slr6095 and Slr6096 are similar to the N-terminal and C-terminal portions of the M subunit (for methylation), respectively (Supplementary Figure S1). Slr6097 is predicted to contain a methylase-S domain and shows high similarity to the S subunit HsdS (for sequence recognition, GenBank accession no. AFWS02000001.1) of a predicted type I RM system in *Thiorhodovibrio* sp. 970 (69% similarity; *e*-value, 4e-108). In the same gene cluster, Slr6012 contains an HSDR_N domain and is similar to the R subunit HsdR (for restriction) in *Thiorhodovibrio* sp. 970 (59% similarity; *e*-value, 0.0). We tentatively call these proteins M. Ssp6803V-n, M. Ssp6803V-c (*n* and *c*, N-terminal, or C-terminal portion), S. Ssp6803V, and R.Ssp6803V.

**FIGURE 2:**
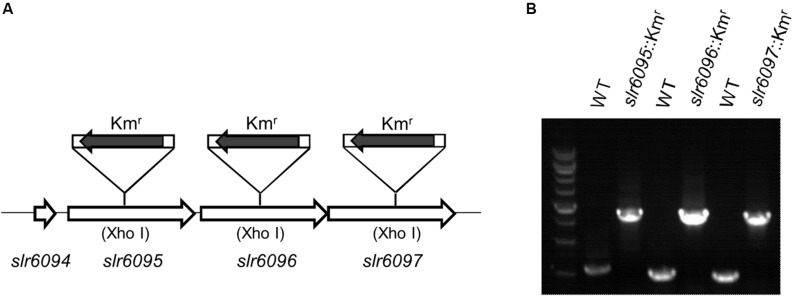
Inactivation of genes in *Synechocystis* 6803. **(A)** Schematic diagram showing insertion of target genes with the Km^r^ cassette. **(B)** Agarose electrophoresis of PCR products for examination of mutants.

To investigate the functions of *slr6095*, *slr6096*, and *slr6097*, we generated mutant strains for each of them (Figure 2A). A Km^r^ cassette was inserted into the target genes via homologous double crossover, and the complete segregation of mutants was confirmed with PCR (Figure 2B). Because M and S subunits of a type I RM system are involved in the restriction activity, the complete segregation of the three mutants does not imply that the predicted R subunit (Slr6102) is not functional.

### Methylome and Transcriptome Analyses

We examined the whole-genome methylation landscape using SMRT sequencing technology. This approach is modestly sensitive to ^m5^C methylation but detects ^m6^A and ^m4^C with high accuracy and sensitivity. As shown in Table 1, in the wild-type genome there are methylations at motifs G^m6^ATC, GG^m4^CC, GA^m6^AGGC, and GG^m6^AN_7_TTGG/CCA^m6^AN_7_TCC. Unlike the wild type, mutant strains *slr6095*::Km^r^, *slr6096*::Km^r^, and *slr6097*::Km^r^ showed no bipartite methylation at GG^m6^AN_7_TTGG/CCA^m6^AN_7_TCC (hereafter Ssp6803V methylation). This is consistent with the predicted functions of the three genes. We also noticed that the GG^m4^CC methylation decreased in *slr6096*::Km^r^ and *slr6097*::Km^r^ in comparison to the wild type level. This could be due to an indirect effect of the two genes on the efficiency of GG^m4^CC methylation.

**TABLE 1:** Detection of DNA methylation in *Synechocystis* 6803 and mutants.

**Motifs for methylation**	**Strains**	**Percentage of methylation**	**Number of methylated motifs**	**Number of predicted motifs**
G^m6^ATC	WT	99.83 ± 0.2	32,863	32,920
	*slr6095*::Km^r^	99.9 ± 0.06	32,886	32,920
	*slr6096*::Km^r^	97.09 ± 2.01	31,962	32,920
	*slr6097*::Km^r^	99.45 ± 0.44	32,739	32,920
GG^m4^CC	WT	98.8 ± 0.36	40,022	40,508
	*slr6095*::Km^r^	90.1 ± 5.03	36,499	40,508
	*slr6096*::Km^r^	57.23 ± 7.88	23,182	40,508
	*slr6097*::Km^r^	72.23 ± 6.25	29,258	40,508
GA^m6^AGGC	WT	99.62 ± 0.44	1446	1451
	*slr6095*::Km^r^	98.97 ± 1.17	1436	1451
	*slr6096*::Km^r^	88.22 ± 5.26	1280	1451
	*slr6097*::Km^r^	95.83 ± 1.9	1391	1451
GG^m6^AN_7_TTGG	WT	99.48 ± 0.17	1255	1261
	*slr6095*::Km^r^	0	0	1261
	*slr6096*::Km^r^	0	0	1261
	*slr6097*::Km^r^	0	0	1261
CCA^m6^AN_7_TCC	WT	99.6 ± 0	1256	1261
	*slr6095*::Km^r^	0	0	1261
	*slr6096*::Km^r^	0	0	1261
	*slr6097*::Km^r^	0	0	1261

DNA methylation may alter gene expression. We performed RNA-seq analyses to compare gene expression in the wild type and mutants (Supplementary Tables S3–S5). Relative to gene expression in the wild type, 143 genes were up-regulated and 160 genes down-regulated in *slr6095*::Km^r^, 137 genes up-regulated, and 172 genes down-regulated in *slr6096*::Km^r^, 109 genes up-regulated, and 150 genes down-regulated in *slr6097*::Km^r^. There are 73 genes up-regulated and 98 genes down-regulated in all three mutants (Table 2). These 171 genes (73 + 98) are involved in photosynthesis, ATP synthesis, pigment syntheses, carbon metabolism, nutrient transport, stress tolerance, signal transduction, and gene regulation, etc.

**TABLE 2:** Ssp6803V methylation and differential expression*.

**Gene**	**Annotation**	TSS site**	**Methylation position**	**Ratio of gene expression**					
				***slr6095*::Km^r^/WT**	***P*-value**	***slr6096*::Km^r^/WT**	***P*-value**	***slr6097*::Km^r^/WT**	***P*-value**
*sll0170* (*dnaK2*)	DnaK protein 2, heat shock protein 70	−20	+6	0.4151	0.0026	0.2577	0.0000	0.3237	0.0007
*sll0199* (*petE*)	Plastocyanin	−98	None	37.0240	0.0000	44.7207	0.0000	48.7945	0.0000
*sll0254* (*crtL*)	Phytoene dehydrogenase Rieske iron-sulfur component	−49	+20, +918, +1253	0.1844	0.0005	0.1729	0.0007	0.1610	0.0003
*sll0416* (*groEL-2*)	60 kDa chaperonin 2, GroEL2	Not ident.	−298	0.2612	0.0003	0.1348	0.0000	0.1730	0.0000
*sll1031* (*ccmM*)	CO_2_ concentrating mechanism protein CcmM	Not ident.	+1191	0.3955	0.0031	0.3169	0.0009	0.3209	0.0027
*sll1281* (*psbZ*)	Photosystem II protein PsbZ	−282	None	0.2460	0.0037	0.2027	0.0007	0.1737	0.0004
*sll1286*	Transcriptional regulator	−27	−257	3.7930	0.0002	4.3482	0.0002	4.1379	0.0004
*sll1325* (*atpD*)	ATP synthase delta chain of CF(1)	Not ident.	None	0.2789	0.0005	0.3369	0.0051	0.2163	0.0004
*sll1327* (*atpC*)	ATP synthase gamma chain	Not ident.	−642	0.3708	0.0043	0.3381	0.0031	0.3022	0.0045
*sll1356* (*glgP*)	Glycogen phosphorylase	−14	+2153, +2285	2.8489	0.0010	3.2206	0.0007	3.6499	0.0005
*sll1382* (*petF*)	Ferredoxin, PetF-like protein	−10	None	0.4459	0.0059	0.3637	0.0038	0.3825	0.0042
*sll1514* (*hspA*)	16.6 kDa small heat shock protein	−41	None	0.0890	0.0000	0.0600	0.0000	0.0491	0.0000
*sll1579* (*cpcC2*)	Phycobilisome rod linker polypeptide	Not ident.	None	0.2747	0.0003	0.1525	0.0000	0.1419	0.0000
*sll1580* (*cpcC1*)	Phycobilisome rod linker polypeptide	Not ident.	None	0.3043	0.0004	0.1843	0.0000	0.1535	0.0000
*sll1883* (*argJ*)	Arginine biosynthesis bifunctional protein ArgJ	−28	−223, +716	0.2984	0.0001	0.3149	0.0013	0.2580	0.0002
*sll1926*	Hypothetical protein	−81	−660, -336, -227	14.0133	0.0000	15.4892	0.0002	12.8917	0.0001
*slr0011* (*rbcX*)	Rubisco chaperonin	Not ident.	None	4.2534	0.0000	3.4429	0.0049	3.9258	0.0000
*slr0056* (*chlG*)	Chlorophyll *a* synthase	Not ident.	+724	0.2575	0.0001	0.2858	0.0007	0.3234	0.0028
*slr0093* (*dnaJ*)	DnaJ protein, heat shock protein 40	−108	−193	0.1370	0.0000	0.1181	0.0000	0.1099	0.0000
*slr0653* (*sigA*)	Principal RNA polymerase sigma factor SigA	−223	+29, +845	4.7465	0.0000	4.5298	0.0000	4.2149	0.0000
*slr0895* (*prqR*)	Transcriptional regulator	Not ident.	None	0.1921	0.0004	0.1458	0.0006	0.1657	0.0001
*slr1185* (*petC2*)	Cytochrome *b*_6_-*f* complex alternative iron-sulfur subunit	Not ident.	None	0.3490	0.0053	0.2335	0.0010	0.2333	0.0048
*slr1285* (*hik34*)	Two-component sensor histidine kinase	Not ident.	None	0.3257	0.0001	0.2269	0.0001	0.1969	0.0000
*slr1291* (*ndhD2*)	NADH dehydrogenase subunit 4	Not ident.	−337, +428	0.2032	0.0000	0.1512	0.0000	0.1420	0.0000
*slr1330* (*atpE*)	ATP synthase epsilon chain of CF1	Not ident.	−680	0.2507	0.0003	0.3063	0.0025	0.2177	0.0005
*slr1655* (*psaL*)	Photosystem I subunit XI	Not ident.	None	0.1807	0.0002	0.1193	0.0000	0.1053	0.0000
*ssl2598* (*psbH*)	Photosystem II protein PsbH	−37	None	0.1785	0.0001	0.1469	0.0000	0.1617	0.0002

* This table includes some of the differentially expressed genes, whereas Supplementary Table S2 includes a full list of the genes. ** not ident., not identified.

To understand the effects of DNA methylation on gene expression in *Synechocystis* 6803, we attempted to find out the relationship between DNA methylation and gene expression. The Ssp6803V methylation sites are distributed in the encoding sequences of 869 genes (23.74% of all in the genome) and the sequences (1 kb) upstream of 270 genes (7.38% of all). Of the 171 genes with changed expression, 75 have no recognition sequence for the Ssp6803V methylation, 40 have the motifs only in their encoding regions (Table 2 and Supplementary Table S2). It is not possible for these genes to be directly regulated by the DNA methylation. However, there are some genes, such as *sll1286* (transcriptional regulator), *sll1883* (*argJ*), *sll1926* (hypothetical protein), and *slr0093* (*dnaJ*), whose Ssp6803V methylation sites may overlap regulatory sequences (see transcription start sites and methylation positions in Table 2). The expression of these genes could be modulated by methylation due to altered interactions between regulatory proteins and recognition sites.

### Effect of DNA Methylation on Glycogen Catabolism

**FIGURE 3:**
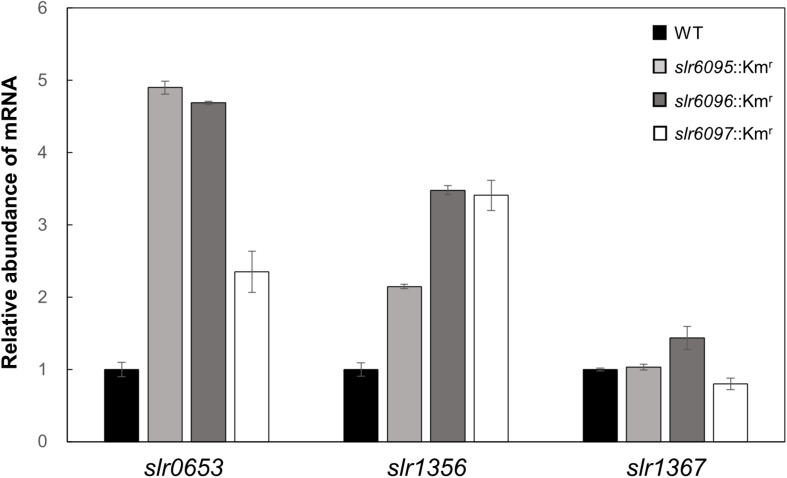
RT-qPCR analysis of gene expression in *Synechocystis* 6803 and mutants.

Among genes that showed changed expression in the mutants relative to the wild type, *slr0653* encodes the principal RNA polymerase sigma factor (SigA), *sll1356* (*glgP1*) encodes GlgP. Both were upregulated in the mutants. RT-qPCR analysis confirmed the increased expression of *sigA* and *glgP1* in three mutants; *glgP2*, however, showed no or slight change in mRNA level (Figure 3).

**FIGURE 4:**
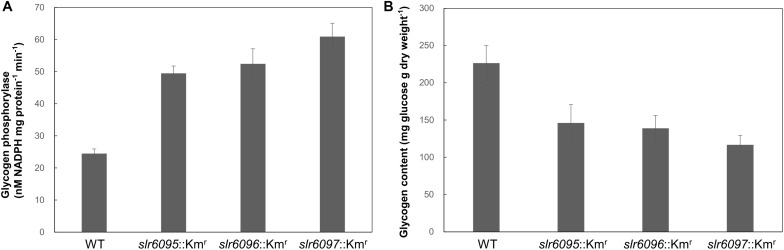
Glycogen phosphorylase activities **(A)** and glycogen contents **(B)** in *Synechocystis* 6803 and mutants.

Because GlgP1 plays an important role in glycogen turnover (Fu and Xu, 2006), we further measured the GlgP activities and total glycogen contents in these strains under photoautotrophic conditions. As shown in Figure 4A, all three mutants showed higher GlgP activities than the wild type. Higher GlgP activity implies more rapid digestion of glycogen. Consistently, the mutants showed lower glycogen contents than the wild type (Figure 4B).

Some genes involved in photosynthesis (phycobilisome, photosystem I, photosystem II, and CO_2_ concentrating mechanism), electron transfer (*petC2*, *petF*), or ATP synthesis were downregulated in the mutants (Table 2), but *petE* and *rbcX* (Rubisco chaperonin) were significantly upregulated. Some heat shock protein/molecular chaperone genes were also downregulated in the mutants (Table 2). The mutants showed no or very slight change in growth at 30°C but reduced growth at 40°C compared to the wild type (Supplementary Figure S2 and data no shown).

## Discussion

In *Synechocystis* 6803, the role of GG^m4^CC methylation in regulation of gene expression has been reported (Gärtner et al., 2019). Now we show that the GG^m6^AN_7_TTGG/CCA^m6^AN_7_TCC methylation, catalyzed by the type I RM system Ssp6803V, is also involved in regulation/modulation of gene expression.

Genes *slr6095*-*slr6096*-*slr6097* and *slr6102* on the plasmid pSYSX are predicted to encode a type I RM system, but the ancestor M subunit gene split into *slr6095* and *slr6096* in the evolution. Unlike a type II RM system, in which the restriction activity is only dependent on the restriction enzyme, cleavage of the target DNA by a type I RM system is dependent on S, R, and M subunits (Murray, 2000). Therefore, successful inactivation of genes for M and S subunits does not imply that the R subunit gene is not functional. In the RNA-seq data, the mRNA abundance of *slr6102* is similar to *slr6095*-*slr6096*-*slr6097*. It is possible that this type I RM system is fully functional.

In *Synechocystis* 6803 mutants *slr6095*::Km^r^, *slr6096*::Km^r^, and *slr6097*::Km^r^, the Ssp6803V methylation was completely eliminated. Downstream of *slr6097* is *sll6098*, which is oriented 3’ to 3’ relative to *slr6097* (Figure 1). Therefore, the phenotype of *slr6097*::Km^r^ could not be attributed to a polar effect. Insertion of *slr6095* and *slr6096* may have polar effects on the downstream gene(s), but these two and *slr6097* are very closely related in function, their phenotypes are supposed to be similar to each other. The very similar phenotypes of the three mutants excluded the possibility that a second mutation might have caused the elimination of DNA methylation and physiological effects, if any.

To analyze the effects of Ssp6803V methylation on gene expression in *Synechocystis* 6803, we compared the mRNA expression profiles of the wild type and mutants and identified 171 genes that showed up- or down-regulated expression in all three mutants. Most of these genes are apparently indirectly regulated by the Ssp6803V methylation (Table 2). *glgP1* is one of those upregulated genes (Table 2 and Figure 3), suggesting that the type I RM system may tune glycogen catabolism via gene regulation. Differences in GlgP activities and glycogen contents between the wild type and mutants (Figure 4) confirmed such a physiological effect of the Ssp6803 methylation. Apparently, the DNA methylation reduces the expression of GlgP1 for glycogen turnover and enhances the storage of glycogen in *Synechocystis* 6803. The mutants also showed reduced growth at 40°C. This phenotype may reflect an integrated effect of changed gene expression (such as expression of genes for photosynthesis, stress responses, etc.) on the high temperature tolerance. In addition, the changed expression of heat shock protein genes (Table 2) could affect salt and oxidative stress tolerance (Asadulghani et al., 2004; Sakthivel et al., 2009; Kim et al., 2014) as well.

The impact of type I RM systems on gene expression has been reported in other bacteria (De Ste Croix et al., 2017). For an example, in *Streptococcus pneumoniae*, a human pathogen, the virulence was shown to be regulated by a type I RM system (SpnD39III) with phase variable recognition specificities (Manso et al., 2014). SpnD39III variants showed distinct genome methylation patterns and gene expression profiles. However, there are no SpnD39III sites in promoters or regulatory regions of those differentially expressed genes. Similarly, no Ssp6803V sites can be found upstream of most genes differentially expressed in the wild type and methylation mutants. Possibly, addition of methyl groups to target sequences can alter protein-DNA interactions that are involved in maintaining regional DNA conformation, therefore indirectly modulates the expression of *glgP1* and many other genes. The underlying mechanism for type I RM systems to impact gene expression in bacteria should be a further question to be tackled in the future.

## Data Availability Statement

Raw sequence read data for DNA methylation analysis has been deposited in the NCBI SRA with the study identifier SRP256040 (runs: SRR11528407-SRR11528414). Raw sequence read data for RNA-seq has been deposited in the NCBI SRA with the study identifier SRP258660 (runs: SRR11614230-SRR11614241).

## Author Contributions

XX designed and supervised the project. DW performed the experiments and bioinformatic analyses. YW performed bioinformatic analyses. XX, DW, and YW analyzed the data. DW and XX wrote the manuscript. XX edited the manuscript.

## Conflict of Interest

The authors declare that the research was conducted in the absence of any commercial or financial relationships that could be construed as a potential conflict of interest.

## Footnotes

1 https://www.pacb.com/support/software-downloads/

2 http://genome.microbedb.jp/cyanobase

3 http://rebase.neb.com/rebase/rebase.html

4 http://genome.microbedb.jp/cyanobase

5 http://smart.embl-heidelberg.de

6 https://blast.ncbi.nlm.nih.gov/Blast.cgi

7 https://www.genome.jp/tools-bin/clustalw
