# Supplementary material for: Effects of a Type I RM System on Gene Expression and Glycogen Catabolism in Synechocystis sp. PCC 6803
Source: Front Microbiol. 2020 Jun 9;11:1258. doi: 10.3389/fmicb.2020.01258 (PMC7296061; doi:10.3389/fmicb.2020.01258)
Supplement: Supplementary file 1 [file Data_Sheet_1.docx]

**Table S1 *Synechocystis* strains, plasmids and primers**

| **Strains** | **Derivation and relevant characteristics *^a^*** | **Reference or source** |
| --- | --- | --- |
| WT | A unicellular cyanobacterium model strain | Zhao J,  Institute of Hydrobiology/Peking Univ. |
| *slr6095*::Km^r^ | Km^r^, *slr6095* mutant, with the Km^r^ cassette inserted into the middle of *slr6095* via double homologous crossover, generated by transformation of the WT with pHB6515 | This study |
| *slr6096*::Km^r^ | Km^r^, *slr6096* mutant, with the Km^r^ cassette inserted into the middle of *slr6096*, generated by transformation with pHB6520 |  |
| *slr6097*::Km^r^ | Km^r^, *slr6097* mutant, with the Km^r^ cassette inserted into the middle of *slr6097*, generated by transformation with pHB6517 |  |
|  | | |
| **Plasmids** | **Derivation and relevant characteristics *^a^*** | **Reference or source** |
| pHB6511 | Ap^r^, the DNA fragment containing *slr6095*, with XhoI site introduced between bp 827 and bp 832, was generated by overlap PCR using primers slr6095-1/R and slr6095-F/2 and cloned into pMD18-T | This study |
| pHB6512 | Ap^r^, the DNA fragment containing *slr6097*, with XhoI site introduced between bp 586 and bp 591, was generated by overlap PCR using primers slr6097-1/R and slr6097-F/2 and cloned into pMD18-T |  |
| pHB6513 | Ap^r^, the DNA fragment containing *slr6096*, with XhoI site introduced between bp 583 and bp 588 , was generated by overlap PCR using primers slr6096-1/R and slr6096-F/2 and cloned into pMD18-T |  |
| pHB6515 | Ap^r^Km^r^, the Km^r^ cassette excised with Bam HI from pRL446, blunted with T4 DNA polymerase, was cloned into Xho I-cut and T4 DNA polymerase-blunted pHB6511, interrupting *slr6095* |  |
| pHB6517 | Ap^r^Km^r^, the Km^r^ cassette excised with Bam HI from pRL446, blunted with T4 DNA polymerase, was cloned into Xho I-cut and T4 DNA polymerase-blunted pHB6512, interrupting *slr6097* |  |
| pHB6520 | Ap^r^Km^r^, the Km^r^ cassette excised with Bam HI from pRL446, blunted with T4 DNA polymerase, was cloned into Xho I-cut and T4 DNA polymerase-blunted pHB6513, interrupting *slr6096* |  |
| pMD18-T | Ap^r^, a cloning vector | Takara, Japan |
| pRL446 | Ap^r^Km^r^, a plasmid containing a Km^r^ cassette | NCBI GenBank  accession no.  EU346690 |
|  | | |
| **Primers** | **Sequences (5′→3′)** | **Reference or source** |
| Slr6095-1 | GGAGTATATCTTTGGG ATGTTATTCCTTAA | This study |
| Slr6095-2 | ATCTCCCTAAGCCTTTTCCA |  |
| Slr6095-F | CATGGCTCGAGGGTGTCCGGAGTCGGGTAAGAAAGCCG |  |
| Slr6095-R | CGGCTTTCTTACCCGACTCCGGACACCCTCGAGCCATG |  |
| 6095-p-1 | ATGTGGTGCAGTTGATGGTGCG |  |
| 6095-p-2 | CCCAGCCCGACGCATCACCA |  |
| Slr6096-1 | GAGATTTTGGTAATGCAATA |  |
| Slr6096-2 | ATTCCAATTATTAGACATATCC |  |
| Slr6096-F | CGGTGAAAAATGTGGCCAAGGCTCGAGTGGACGATCGCCGGAAGG |  |
| Slr6096-R | GACCTTCCGGCGATCGTCCACTCGAGCCTTGGCCACATTTTTCACCG |  |
| 6096-p-1 | GATGAGGTACGGTATGAGTTGC |  |
| 6096-p-2 | GTTATAGGGTTCCAACCTCGCC |  |
| Slr6097-1 | TTGGAGGGATTGGGATATGTCTAAT |  |
| Slr6097-2 | GCGCCCTATTTAGCGGTTTTTA |  |
| Slr6097-F | GATCGGGCTTATTCCTCGAGAAGGAGTGGGATA |  |
| Slr6097-R | TATCCCACTCCTTCTCGAGGAATAAGCCCGATC |  |
| 6097-p-1 | TAGTTCTACAGGAGTACCTGGAC |  |
| 6097-p-2 | TACTGCAATTGTTCCTACAGGA |  |
| RT-*rnpB*-1 | CGGTTGGAAGCAAGGTCG |  |
| RT-*rnpB*-2 | GTCGTAAGCCGGGTTCTG |  |
| RT-*slr0653*-1 | CGCCGAGCCAAAGACAAAAT |  |
| RT-*slr0653*-2 | GTTTCGTAGAGGTGGACGGG |  |
| RT-*sll1356*-1 | CCATACGGAACCCTACACCG |  |
| RT-*sll1356*-2 | CTGCCCCGTAATAATCCCCC |  |
| RT-*slr1367*-1 | TTGCGCCTAGCCCAACAATA |  |
| RT-*slr1367*-2 | AAACAGGCGCATCAATTCGG |  |

*^a^* Ap, ampicillin; Km, kanamycin; unless stated otherwise, the template for PCR reactions was *Synechocystis* sp. genomic DNA.

**Table S2 Ssp6803V methylation and differential expression**

| **ID** | **gene** | **annotation** | **TSS site** | **Methylation position** | **Ratio of gene expression** | | | | | |
| --- | --- | --- | --- | --- | --- | --- | --- | --- | --- | --- |
|  |  |  |  |  | ***slr6095*::Km^r^/WT** | **P-**  **value** | ***slr6096*::Km^r^/WT** | **P-**  **value** | ***slr6097*::Km^r^/WT** | **P- value** |
| *sll0048* |  | unknown protein | -96 | -95 | 0.3638 | 0.0009 | 0.3505 | 0.0038 | 0.3567 | 0.0056 |
| *sll0148* |  | hypothetical protein | -28 | none | 0.3781 | 0.0024 | 0.2962 | 0.0007 | 0.2869 | 0.0012 |
| *sll0170* | *dnaK2* | DnaK protein 2, heat shock protein 70, molecular chaperone | -20 | +6 | 0.4151 | 0.0026 | 0.2577 | 0.0000 | 0.3237 | 0.0007 |
| *sll0185* |  | hypothetical protein | not identified | +1107 | 0.3348 | 0.0002 | 0.3069 | 0.0002 | 0.2532 | 0.0001 |
| *sll0199* | *petE* | plastocyanin | -98 | none | 37.0240 | 0.0000 | 44.7207 | 0.0000 | 48.7945 | 0.0000 |
| *sll0254* | *crtL* | probable phytoene dehydrogenase Rieske iron-sulfur component | -49 | +20, +918, +1253 | 0.1844 | 0.0005 | 0.1729 | 0.0007 | 0.1610 | 0.0003 |
| *sll0382* |  | hypothetical protein | not identified | none | 0.2654 | 0.0001 | 0.3623 | 0.0041 | 0.3683 | 0.0081 |
| *sll0408* |  | peptidyl-prolyl cis-trans isomerase | not identified | +449 | 0.4067 | 0.0045 | 0.3443 | 0.0019 | 0.2821 | 0.0011 |
| *sll0416* | *groEL-2* | 60 kDa chaperonin 2, GroEL2, molecular chaperone | not identified | -298 | 0.2612 | 0.0003 | 0.1348 | 0.0000 | 0.1730 | 0.0000 |
| *sll0446* |  | unknown protein | not identified | none | 2.8991 | 0.0003 | 3.0485 | 0.0007 | 2.6504 | 0.0047 |
| *sll0481* |  | unknown protein | not identified | none | 0.3010 | 0.0044 | 0.2669 | 0.0020 | 0.2438 | 0.0022 |
| *sll0482* |  | unknown protein | not identified | +264, +857 | 0.2953 | 0.0004 | 0.2249 | 0.0001 | 0.1579 | 0.0000 |
| *sll0520* | *ndhI* | NADH dehydrogenase subunit NdhI | not identified | +112 | 4.2378 | 0.0001 | 6.1593 | 0.0001 | 5.1546 | 0.0001 |
| *sll0528* |  | hypothetical protein | -7 | none | 0.2966 | 0.0051 | 0.1949 | 0.0013 | 0.2289 | 0.0016 |
| *sll0535* | *clpX* | ATP-dependent Clp protease ATPase subunit | not identified | +8 | 2.9815 | 0.0016 | 3.0563 | 0.0025 | 2.9385 | 0.0060 |
| *sll0543* |  | hypothetical protein | not identified | none | 4.2503 | 0.0000 | 6.7936 | 0.0000 | 3.2132 | 0.0019 |
| *sll0594* |  | transcriptional regulator | not identified | -89 | 0.2808 | 0.0000 | 0.2135 | 0.0000 | 0.2795 | 0.0001 |
| *sll0595* |  | unknown protein | -24 | -777 | 0.2170 | 0.0036 | 0.2249 | 0.0076 | 0.3087 | 0.0094 |
| *sll0630* |  | unknown protein | not identified | none | 0.1794 | 0.0008 | 0.1633 | 0.0017 | 0.1424 | 0.0012 |
| *sll0680* | *pstS* | phosphate-binding periplasmic protein precursor (PBP) | not identified | none | 0.0733 | 0.0000 | 0.3588 | 0.0022 | 0.0715 | 0.0000 |
| *sll0681* | *pstC* | phosphate transport system permease protein PstC homolog | not identified | none | 0.0256 | 0.0000 | 0.2808 | 0.0003 | 0.0248 | 0.0000 |
| *sll0682* | *pstA* | phosphate transport system permease protein PstA homolog | not identified | none | 0.0356 | 0.0000 | 0.2395 | 0.0002 | 0.0357 | 0.0000 |
| *sll0726* | *pgm* | phosphoglucomutase | -43 | none | 0.2540 | 0.0001 | 0.2036 | 0.0000 | 0.2587 | 0.0005 |
| *sll0767* | *rpl20* | 50S ribosomal protein L20 | -3 | -51 | 2.7525 | 0.0012 | 2.9071 | 0.0023 | 2.5550 | 0.0085 |
| *sll0798* | *nrsS* | Ni(II)-sensor and/or redox sensor, two-component sensor histidine kinase | not identified | +1292 | 0.3291 | 0.0079 | 0.2877 | 0.0050 | 0.2317 | 0.0015 |
| *sll0830* | *fus* | elongation factor EF-G | not identified | +671, +1272, +1939 | 2.6100 | 0.0009 | 2.5326 | 0.0049 | 2.4540 | 0.0094 |
| *sll0844* |  | tRNA (5-methylaminomethyl-2-thiouridylate)-methyltransferase | -56 | +438 | 0.2828 | 0.0001 | 0.2155 | 0.0000 | 0.2082 | 0.0000 |
| *sll0846* |  | hypothetical protein | -17 | -50 | 0.4807 | 0.0041 | 0.3284 | 0.0006 | 0.2494 | 0.0000 |
| *sll0872* |  | unknown protein | not identified | none | 3.3987 | 0.0009 | 3.1898 | 0.0024 | 3.6022 | 0.0018 |
| *sll0938* |  | aspartate transaminase | not identified | none | 0.1417 | 0.0000 | 0.1031 | 0.0000 | 0.1173 | 0.0000 |
| *sll0939* |  | hypothetical protein | not identified | +203 | 0.2598 | 0.0033 | 0.1850 | 0.0021 | 0.1987 | 0.0013 |
| *sll0982* |  | unknown protein | not identified | -709, -147 | 0.1754 | 0.0007 | 0.2293 | 0.0051 | 0.2575 | 0.0060 |
| *sll1024* |  | hypothetical protein | not identified | -997 | 0.3332 | 0.0016 | 0.3150 | 0.0011 | 0.3371 | 0.0037 |
| *sll1025* |  | hypothetical protein | not identified | -404, +462, +684 | 0.3037 | 0.0002 | 0.2618 | 0.0001 | 0.3253 | 0.0018 |
| *sll1031* | *ccmM* | carbon dioxide concentrating mechanism protein CcmM, putative carboxysome structural protein | not identified | +1191 | 0.3955 | 0.0031 | 0.3169 | 0.0009 | 0.3209 | 0.0027 |
| *sll1086* |  | unknown protein | not identified | none | 0.2636 | 0.0000 | 0.1923 | 0.0000 | 0.2432 | 0.0001 |
| *sll1127* | *menB* | 1,4-dihydroxy-2-naphthoate synthase | not identified | -68 | 0.2484 | 0.0000 | 0.1833 | 0.0000 | 0.2250 | 0.0000 |
| *sll1181* | *hlyD* | similar to hemolysin secretion protein | -82,-61 | -755, +1047 | 3.3634 | 0.0001 | 3.6473 | 0.0001 | 3.5899 | 0.0003 |
| *sll1214* | *ycf59* | hypothetical protein YCF59 | not identified | -765 | 5.1227 | 0.0000 | 4.6210 | 0.0000 | 4.5587 | 0.0000 |
| *sll1222* |  | hypothetical protein | not identified | none | 3.5510 | 0.0001 | 5.2791 | 0.0000 | 2.7585 | 0.0043 |
| *sll1234* | *ahcY* | adenosylhomocysteinase | -31 | +998 | 0.2966 | 0.0002 | 0.2354 | 0.0001 | 0.2307 | 0.0002 |
| *sll1236* |  | unknown protein | -37 | +706 | 13.0902 | 0.0025 | 16.6840 | 0.0000 | 22.0361 | 0.0018 |
| *sll1254* |  | hypothetical protein | -20 | -519, +119 | 3.9147 | 0.0011 | 3.2527 | 0.0071 | 4.3575 | 0.0014 |
| *sll1281* | *psbZ* | photosystem II PsbZ protein | -282 | none | 0.2460 | 0.0037 | 0.2027 | 0.0007 | 0.1737 | 0.0004 |
| *sll1286* |  | transcriptional regulator | -27 | -257 | 3.7930 | 0.0002 | 4.3482 | 0.0002 | 4.1379 | 0.0004 |
| *sll1325* | *atpD* | ATP synthase delta chain of CF(1) | not identified | none | 0.2789 | 0.0005 | 0.3369 | 0.0051 | 0.2163 | 0.0004 |
| *sll1327* | *atpC* | ATP synthase gamma chain | not identified | -642 | 0.3708 | 0.0043 | 0.3381 | 0.0031 | 0.3022 | 0.0045 |
| *sll1356* | *glgP* | glycogen phosphorylase | -14 | +2153, +2285 | 2.8489 | 0.0010 | 3.2206 | 0.0007 | 3.6499 | 0.0005 |
| *sll1382* | *petF* | ferredoxin, *petF*-like protein | -10 | none | 0.4459 | 0.0059 | 0.3637 | 0.0038 | 0.3825 | 0.0042 |
| *sll1414* |  | hypothetical protein | -25 | -619, +1573 | 2.9088 | 0.0029 | 3.2740 | 0.0011 | 2.9121 | 0.0050 |
| *sll1434* | *mrcA* | penicillin-binding protein | not identified | none | 0.4401 | 0.0050 | 0.3425 | 0.0029 | 0.3632 | 0.0033 |
| *sll1483* |  | periplasmic protein, similar to transforming growth factor induced protein | not identified | none | 0.2950 | 0.0000 | 0.1820 | 0.0000 | 0.2235 | 0.0001 |
| *sll1514* | *hspA* | 16.6 kDa small heat shock protein, molecular chaperone | -41 | none | 0.0890 | 0.0000 | 0.0600 | 0.0000 | 0.0491 | 0.0000 |
| *sll1541* |  | hypothetical protein | not identified | -723 | 0.1489 | 0.0000 | 0.1806 | 0.0000 | 0.1788 | 0.0000 |
| *sll1564* | *leuA* | putative lyase | not identified | -918, +11, +197, +639 | 0.4260 | 0.0044 | 0.3518 | 0.0033 | 0.3845 | 0.0072 |
| *sll1579* | *cpcC2* | phycobilisome rod linker polypeptide | not identified | none | 0.2747 | 0.0003 | 0.1525 | 0.0000 | 0.1419 | 0.0000 |
| *sll1580* | *cpcC1* | phycobilisome rod linker polypeptide | not identified | none | 0.3043 | 0.0004 | 0.1843 | 0.0000 | 0.1535 | 0.0000 |
| *sll1583* |  | unknown protein | -55 | +275 | 4.3109 | 0.0000 | 4.2089 | 0.0000 | 5.2358 | 0.0000 |
| *sll1641* | *gad* | glutamate decarboxylase | not identified | +1151 | 0.3823 | 0.0046 | 0.3348 | 0.0030 | 0.3319 | 0.0038 |
| *sll1654* |  | hypothetical protein | not identified | -610, +454 | 0.3623 | 0.0009 | 0.2306 | 0.0000 | 0.3364 | 0.0014 |
| *sll1655* | *birA* | similar to biotin [acetyl-CoA-carboxylase] ligase | not identified | +25 | 0.3403 | 0.0003 | 0.2257 | 0.0000 | 0.3103 | 0.0003 |
| *sll1702* | *ycf51* | hypothetical protein YCF51 | -16 | -597 | 0.2282 | 0.0000 | 0.2007 | 0.0000 | 0.2243 | 0.0001 |
| *sll1703* | *sppA1* | protease IV | not identified | +803, +1361 | 0.3878 | 0.0040 | 0.3287 | 0.0031 | 0.3527 | 0.0034 |
| *sll1766* |  | hypothetical protein | not identified | none | 2.6026 | 0.0048 | 3.1402 | 0.0013 | 3.0027 | 0.0034 |
| *sll1784* |  | periplasmic protein, function unknown | not identified | none | 2.5508 | 0.0090 | 4.3771 | 0.0000 | 3.3730 | 0.0005 |
| *sll1796* | *petJ* | cytochrome c553 | -5 | none | 0.0220 | 0.0000 | 0.0170 | 0.0000 | 0.0154 | 0.0000 |
| *sll1797* | *ycf21* | hypothetical protein YCF21 | not identified | -422 | 0.3084 | 0.0069 | 0.2231 | 0.0037 | 0.2379 | 0.0030 |
| *sll1805* | *rpl16* | 50S ribosomal protein L16 | not identified | none | 2.9894 | 0.0009 | 3.0914 | 0.0022 | 3.5741 | 0.0018 |
| *sll1848* | *plsC* | putative acyltransferas | not identified | none | 0.1955 | 0.0011 | 0.2924 | 0.0096 | 0.0958 | 0.0000 |
| *sll1852* | *ndk* | nucleoside diphosphate kinase | -46 | none | 0.3335 | 0.0030 | 0.1380 | 0.0000 | 0.2315 | 0.0002 |
| *sll1883* | *argJ* | arginine biosynthesis bifunctional protein ArgJ | -28 | -223, +716 | 0.2984 | 0.0001 | 0.3149 | 0.0013 | 0.2580 | 0.0002 |
| *sll1926* |  | hypothetical protein | -81 | -660, -336,  -227 | 14.0133 | 0.0000 | 15.4892 | 0.0002 | 12.8917 | 0.0001 |
| *sll1942* |  | unknown protein | -1 | none | 4.4087 | 0.0095 | 4.7356 | 0.0003 | 4.9279 | 0.0005 |
| *sll7063* |  | unknown protein | not identified | +226 | 6.4783 | 0.0000 | 8.6769 | 0.0000 | 8.2487 | 0.0025 |
| *sll7064* |  | unknown protein | not identified | none | 11.8060 | 0.0000 | 13.8173 | 0.0000 | 13.8868 | 0.0010 |
| *sll7066* |  | unknown protein | not identified | +1040, +1152, +1722 | 21.0233 | 0.0000 | 26.5730 | 0.0000 | 25.3103 | 0.0067 |
| *sll7085* |  | unknown protein | not identified | +494 | 4.3227 | 0.0098 | 7.3713 | 0.0000 | 4.3961 | 0.0045 |
| *sll7086* |  | unknown protein | not identified | none | 10.0121 | 0.0003 | 16.3915 | 0.0000 | 11.1372 | 0.0001 |
| *sll7089* |  | unknown protein | not identified | -994, -592, +671 | 4.1405 | 0.0005 | 5.4744 | 0.0000 | 3.9463 | 0.0050 |
| *sll7090* |  | unknown protein | not identified | -624, -549,  -15, +483, +1800, +2128,  +2530 | 8.1951 | 0.0000 | 9.3795 | 0.0000 | 6.6696 | 0.0001 |
| *sll8002* |  | hypothetical protein | not identified | none | 0.2385 | 0.0001 | 0.2642 | 0.0004 | 0.2433 | 0.0008 |
| *sll8034* |  | 2-nitropropane dioxygenase | not identified | -687, -506,  -133 | 0.2581 | 0.0045 | 0.2042 | 0.0003 | 0.2779 | 0.0032 |
| *slr0011* | *rbcX* | possible Rubisco chaperonin | not identified | none | 4.2534 | 0.0000 | 3.4429 | 0.0049 | 3.9258 | 0.0000 |
| *slr0056* | *chlG* | chlorophyll a synthase | not identified | +724 | 0.2575 | 0.0001 | 0.2858 | 0.0007 | 0.3234 | 0.0028 |
| *slr0093* | *dnaJ* | DnaJ protein, heat shock protein 40, molecular chaperone | -108 | -193 | 0.1370 | 0.0000 | 0.1181 | 0.0000 | 0.1099 | 0.0000 |
| *slr0095* |  | O-methyltransferase | not identified | none | 0.2017 | 0.0038 | 0.1536 | 0.0013 | 0.2211 | 0.0022 |
| *slr0103* |  | unknown protein | not identified | none | 4.2770 | 0.0003 | 3.5932 | 0.0014 | 4.3170 | 0.0006 |
| *slr0271* |  | unknown protein | not identified | +951 | 0.1364 | 0.0000 | 0.2240 | 0.0000 | 0.1852 | 0.0000 |
| *slr0272* |  | unknown protein | not identified | -38 | 0.0914 | 0.0000 | 0.1796 | 0.0000 | 0.1462 | 0.0000 |
| *slr0273* |  | unknown protein | not identified | -706, +615 | 0.2083 | 0.0004 | 0.2600 | 0.0031 | 0.1803 | 0.0001 |
| *slr0394* | *pgk* | phosphoglycerate kinase | not identified | -87 | 0.3202 | 0.0004 | 0.2482 | 0.0001 | 0.2656 | 0.0004 |
| *slr0453* |  | hypothetical protein | not identified | -428, -177 | 0.3765 | 0.0013 | 0.3639 | 0.0031 | 0.3530 | 0.0032 |
| *slr0474* | *rcp1* | two-component response regulator CheY subfamily, regulator for phytochrome 1 (Cph1) | not identified | none | 4.6322 | 0.0000 | 4.6832 | 0.0000 | 3.8711 | 0.0003 |
| *slr0476* |  | unknown protein | -145 | none | 0.3262 | 0.0002 | 0.2510 | 0.0000 | 0.2688 | 0.0003 |
| *slr0489* |  | unknown protein | not identified | none | 4.4728 | 0.0002 | 4.0274 | 0.0004 | 5.4110 | 0.0001 |
| *slr0579* |  | unknown protein | not identified | -441 | 0.4134 | 0.0023 | 0.2736 | 0.0002 | 0.2843 | 0.0004 |
| *slr0597* | *purH* | phosphoribosyl aminoimidazole carboxy formyl formyltransferase/inosine  monophosphate cyclohydrolase (PUR-H(J)) | -34 | +614 | 0.3031 | 0.0002 | 0.2199 | 0.0000 | 0.2252 | 0.0000 |
| *slr0617* |  | unknown protein | not identified | none | 0.3466 | 0.0019 | 0.2988 | 0.0013 | 0.2599 | 0.0010 |
| *slr0623* | *trxA* | thioredoxin | -28 | none | 4.0765 | 0.0000 | 3.1057 | 0.0011 | 3.5535 | 0.0006 |
| *slr0653* | *sigA* | principal RNA polymerase sigma factor SigA | -223 | +29, +845 | 4.7465 | 0.0000 | 4.5298 | 0.0000 | 4.2149 | 0.0000 |
| *slr0689* |  | hypothetical protein | not identified | none | 0.3218 | 0.0024 | 0.3459 | 0.0078 | 0.3113 | 0.0043 |
| *slr0708* |  | periplasmic protein, function unknown | not identified | none | 3.5202 | 0.0001 | 3.8168 | 0.0002 | 3.0509 | 0.0037 |
| *slr0787* |  | hypothetical protein | not identified | none | 0.1411 | 0.0001 | 0.1598 | 0.0006 | 0.2287 | 0.0008 |
| *slr0852* |  | hypothetical protein | -35 | +245 | 0.2873 | 0.0018 | 0.2423 | 0.0016 | 0.2464 | 0.0007 |
| *slr0895* | *prqR* | transcriptional regulator | not identified | none | 0.1921 | 0.0004 | 0.1458 | 0.0006 | 0.1657 | 0.0001 |
| *slr0897* | *ssgLC* | probable endoglucanase | not identified | -398, +2590 | 0.2930 | 0.0000 | 0.2220 | 0.0000 | 0.2974 | 0.0004 |
| *slr0967* |  | hypothetical protein | not identified | -520, -327 | 0.3088 | 0.0016 | 0.2018 | 0.0006 | 0.2177 | 0.0003 |
| *slr0989* |  | hypothetical protein | not identified | -37 | 0.1983 | 0.0000 | 0.1536 | 0.0000 | 0.2026 | 0.0002 |
| *slr1028* |  | unknown protein | -13 | +5147, +6246, +7978, +11099 | 3.1777 | 0.0001 | 3.0267 | 0.0008 | 2.4656 | 0.0073 |
| *slr1079* |  | unknown protein | not identified | none | 2.8230 | 0.0018 | 2.6855 | 0.0074 | 3.3217 | 0.0020 |
| *slr1081* |  | hypothetical protein | not identified | none | 3.0174 | 0.0005 | 3.0280 | 0.0012 | 3.7260 | 0.0002 |
| *slr1082* |  | unknown protein | not identified | none | 2.8267 | 0.0010 | 2.9396 | 0.0024 | 3.8109 | 0.0003 |
| *slr1083* |  | hypothetical protein | not identified | none | 3.7873 | 0.0001 | 5.2592 | 0.0001 | 5.3460 | 0.0000 |
| *slr1109* | *ank* | similar to ankyrin | not identified | none | 0.1973 | 0.0000 | 0.2293 | 0.0001 | 0.2461 | 0.0004 |
| *slr1169* |  | unknown protein | not identified | none | 3.4295 | 0.0026 | 3.3182 | 0.0037 | 4.2129 | 0.0046 |
| *slr1173* |  | hypothetical protein | not identified | none | 3.2100 | 0.0018 | 4.3284 | 0.0001 | 5.6683 | 0.0049 |
| *slr1185* | *petC2* | cytochrome b6-f complex alternative iron-sulfur subunit (Rieske iron sulfur protein) | not identified | none | 0.3490 | 0.0053 | 0.2335 | 0.0010 | 0.2333 | 0.0048 |
| *slr1218* | *ycf39* | hypothetical protein YCF39 | not identified | none | 3.9952 | 0.0078 | 5.6079 | 0.0003 | 5.3484 | 0.0014 |
| *slr1240* |  | unknown protein | not identified | none | 5.8910 | 0.0000 | 7.9138 | 0.0000 | 5.3644 | 0.0000 |
| *slr1248* | *pstC* | phosphate transport system permease protein PstC homolog | not identified | none | 0.3019 | 0.0001 | 0.2379 | 0.0000 | 0.2318 | 0.0000 |
| *slr1249* | *pstA* | phosphate transport system permease protein PstA homolog | not identified | none | 0.3936 | 0.0036 | 0.3038 | 0.0010 | 0.3566 | 0.0031 |
| *slr1285* | *hik34* | two-component sensor histidine kinase | not identified | none | 0.3257 | 0.0001 | 0.2269 | 0.0001 | 0.1969 | 0.0000 |
| *slr1291* | *ndhD2* | NADH dehydrogenase subunit 4 | not identified | -337, +428 | 0.2032 | 0.0000 | 0.1512 | 0.0000 | 0.1420 | 0.0000 |
| *slr1330* | *atpE* | ATP synthase epsilon chain of CF(1) | not identified | -680 | 0.2507 | 0.0003 | 0.3063 | 0.0025 | 0.2177 | 0.0005 |
| *slr1376* |  | hypothetical protein | -154 | +107 | 2.6460 | 0.0077 | 2.6983 | 0.0071 | 3.2228 | 0.0034 |
| *slr1394* |  | hypothetical protein | not identified | -763 | 0.2503 | 0.0008 | 0.2488 | 0.0011 | 0.1981 | 0.0003 |
| *slr1413* |  | hypothetical protein | not identified | none | 0.3674 | 0.0007 | 0.3454 | 0.0006 | 0.3283 | 0.0012 |
| *slr1437* |  | unknown protein | not identified | none | 2.6182 | 0.0014 | 2.5518 | 0.0042 | 2.9773 | 0.0027 |
| *slr1444* |  | hypothetical protein | not identified | -52 | 3.4297 | 0.0001 | 3.8443 | 0.0009 | 4.0876 | 0.0001 |
| *slr1452* | *sbpA* | sulfate transport system substrate-binding protein | not identified | -316 | 0.1073 | 0.0034 | 0.0758 | 0.0014 | 0.0926 | 0.0015 |
| *slr1583* |  | hypothetical protein | not identified | -895 | 3.8608 | 0.0000 | 4.3023 | 0.0000 | 3.9513 | 0.0002 |
| *slr1603* |  | hypothetical protein | not identified | none | 0.2663 | 0.0001 | 0.1885 | 0.0000 | 0.1722 | 0.0000 |
| *slr1611* |  | hypothetical protein | -28 | +388 | 3.8633 | 0.0004 | 3.5841 | 0.0011 | 4.6085 | 0.0002 |
| *slr1655* | *psaL* | photosystem I subunit XI | not identified | none | 0.1807 | 0.0002 | 0.1193 | 0.0000 | 0.1053 | 0.0000 |
| *slr1657* |  | hypothetical protein | not identified | none | 4.4676 | 0.0012 | 6.5409 | 0.0001 | 4.1846 | 0.0041 |
| *slr1665* | *dapF* | diaminopimelate epimerase | not identified | +428 | 0.3681 | 0.0055 | 0.2897 | 0.0034 | 0.3029 | 0.0023 |
| *slr1672* | *glpK* | glycerol kinase | not identified | +914, +1344 | 0.3677 | 0.0021 | 0.3693 | 0.0056 | 0.2864 | 0.0010 |
| *slr1712* |  | hypothetical protein | not identified | none | 4.8515 | 0.0026 | 3.3977 | 0.0027 | 4.5838 | 0.0004 |
| *slr1722* | *guaB* | inosine-5'-monophosphate dehydrogenase | not identified | -626，-265 | 0.3990 | 0.0032 | 0.2837 | 0.0007 | 0.3653 | 0.0067 |
| *slr1805* | *hik16* | two-component sensor histidine kinase | not identified | -414, +851,  +1211 | 0.1810 | 0.0002 | 0.1235 | 0.0001 | 0.1480 | 0.0001 |
| *slr1815* |  | hypothetical protein | not identified | +307 | 3.7293 | 0.0009 | 3.5770 | 0.0014 | 3.8013 | 0.0021 |
| *slr1867* | *trpD* | anthranilate phosphoribosyltransferase | not identified | none | 0.2943 | 0.0020 | 0.2331 | 0.0002 | 0.2781 | 0.0013 |
| *slr1895* |  | hypothetical protein | not identified | +456 | 0.3531 | 0.0014 | 0.2718 | 0.0006 | 0.2536 | 0.0008 |
| *slr1906* |  | hypothetical protein | -28 | +231, +419 | 6.5950 | 0.0000 | 4.8756 | 0.0006 | 5.0993 | 0.0012 |
| *slr1917* |  | hypothetical protein | -29 | -512, +656 | 0.3987 | 0.0087 | 0.2653 | 0.0007 | 0.3510 | 0.0059 |
| *slr1924* | *pbp7* | D-alanyl-D-alanine carboxypeptidase, periplasmic protein | not identified | -684 | 3.2929 | 0.0037 | 5.6212 | 0.0000 | 3.6451 | 0.0032 |
| *slr1944* |  | periplasmic protein, function unknown | not identified | -17 | 2.5248 | 0.0026 | 3.1161 | 0.0010 | 2.8708 | 0.0032 |
| *slr2017* | *pilA11* | type 4 pilin-like protein, essential for motility | not identified | none | 2.8610 | 0.0005 | 3.7510 | 0.0001 | 3.6524 | 0.0003 |
| *slr2046* |  | unknown protein | not identified | +2325, +2631, +2937, +3243, +4161, +4467,  +5079 | 2.9221 | 0.0001 | 2.7441 | 0.0015 | 2.8705 | 0.0016 |
| *slr2073* |  | hypothetical protein YCF50 | not identified | none | 2.9567 | 0.0005 | 4.3725 | 0.0000 | 4.0718 | 0.0002 |
| *slr2135* | *hupE* | hydrogenase accessory protein HupE | not identified | none | 0.1349 | 0.0000 | 0.1690 | 0.0000 | 0.1714 | 0.0001 |
| *slr5051* |  | unknown protein | not identified | -442 | 4.3830 | 0.0007 | 4.2291 | 0.0006 | 5.6272 | 0.0001 |
| *slr5119* |  | hypothetical protein | not identified | -801, -697,  -317, +542 | 3.1738 | 0.0004 | 2.4949 | 0.0068 | 3.2244 | 0.0013 |
| *slr6012* |  | unknown protein | not identified | -589, +230 | 6.1121 | 0.0000 | 5.7402 | 0.0000 | 5.3169 | 0.0000 |
| *slr6016* |  | unknown protein | not identified | +297, +1897 | 6.6932 | 0.0000 | 8.3367 | 0.0000 | 6.4649 | 0.0062 |
| *slr6047* |  | hypothetical protein | not identified | -595, -357,  +2135 | 3.2382 | 0.0002 | 3.8930 | 0.0001 | 3.8269 | 0.0033 |
| *slr6071* |  | unknown protein | not identified | -589, -238,  +16, +978 | 6.1121 | 0.0000 | 5.7402 | 0.0000 | 5.3169 | 0.0000 |
| *slr6075* |  | unknown protein | not identified | +201, +561,  +819, +1545,  +2124 | 6.6932 | 0.0000 | 8.3367 | 0.0000 | 6.4649 | 0.0062 |
| *slr7088* |  | hypothetical protein | not identified | +839 | 5.8190 | 0.0032 | 7.3135 | 0.0000 | 4.5254 | 0.0007 |
| *slr8038* |  | WD-repeat protein | not identified | -357, +1212,  +1250, +1665, +2169, +3307,  +3603 | 0.1983 | 0.0000 | 0.2012 | 0.0001 | 0.1464 | 0.0000 |
| *ssl1784* | *rps15* | 30S ribosomal protein S15 | -53 | none | 3.7317 | 0.0029 | 4.3407 | 0.0009 | 4.1593 | 0.0028 |
| *ssl2598* | *psbH* | photosystem II PsbH protein | -37 | none | 0.1785 | 0.0001 | 0.1469 | 0.0000 | 0.1617 | 0.0002 |
| *ssl3335* | *secE* | preprotein translocase SecE subunit | not identified | none | 6.5039 | 0.0000 | 5.7084 | 0.0000 | 5.9159 | 0.0054 |
| *ssl3364* | *cp12* | CP12 polypeptide | -3 | none | 0.2116 | 0.0027 | 0.1564 | 0.0014 | 0.1330 | 0.0005 |
| *ssl3436* | *rpl29* | 50S ribosomal protein L29 | not identified | +90 | 3.4902 | 0.0003 | 3.3387 | 0.0014 | 3.6729 | 0.0089 |
| *ssl3437* | *rps17* | 30S ribosomal protein S17 | not identified | -140 | 2.5492 | 0.0031 | 2.6352 | 0.0051 | 2.8372 | 0.0038 |
| *ssr2194* |  | unknown protein | not identified | +17 | 42.3051 | 0.0001 | 67.7270 | 0.0000 | 75.2272 | 0.0000 |
| *ssr2227* |  | putative transposase | not identified | +106 | 0.3048 | 0.0009 | 0.2301 | 0.0001 | 0.3194 | 0.0036 |
| *ssr2799* | *rpl27* | 50S ribosomal protein L27 | not identified | none | 0.3485 | 0.0021 | 0.2334 | 0.0005 | 0.3094 | 0.0014 |
| *ssr3188* |  | hypothetical protein | -7 | -685, +140 | 0.2221 | 0.0051 | 0.1936 | 0.0032 | 0.2006 | 0.0026 |
| *ssr6048* |  | unknown protein | not identified | none | 4.4314 | 0.0007 | 4.5606 | 0.0006 | 4.8859 | 0.0009 |
