# Supplementary figures and images for: Effects of a Type I RM System on Gene Expression and Glycogen Catabolism in Synechocystis sp. PCC 6803
Source: Front Microbiol. 2020 Jun 9;11:1258. doi: 10.3389/fmicb.2020.01258 (PMC7296061; doi:10.3389/fmicb.2020.01258)

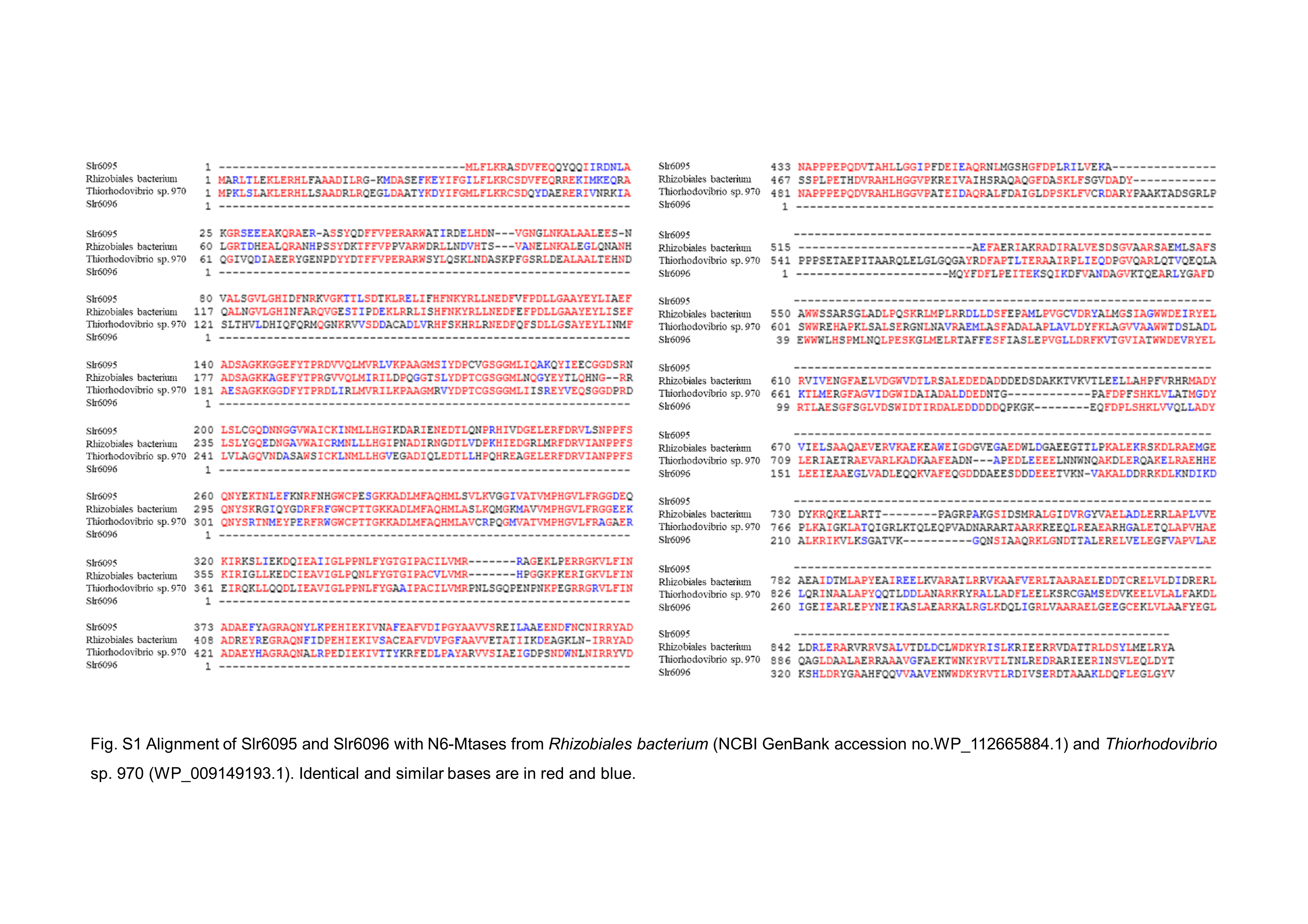

Supplement: Supplementary file 3 [file Image_1.TIF]

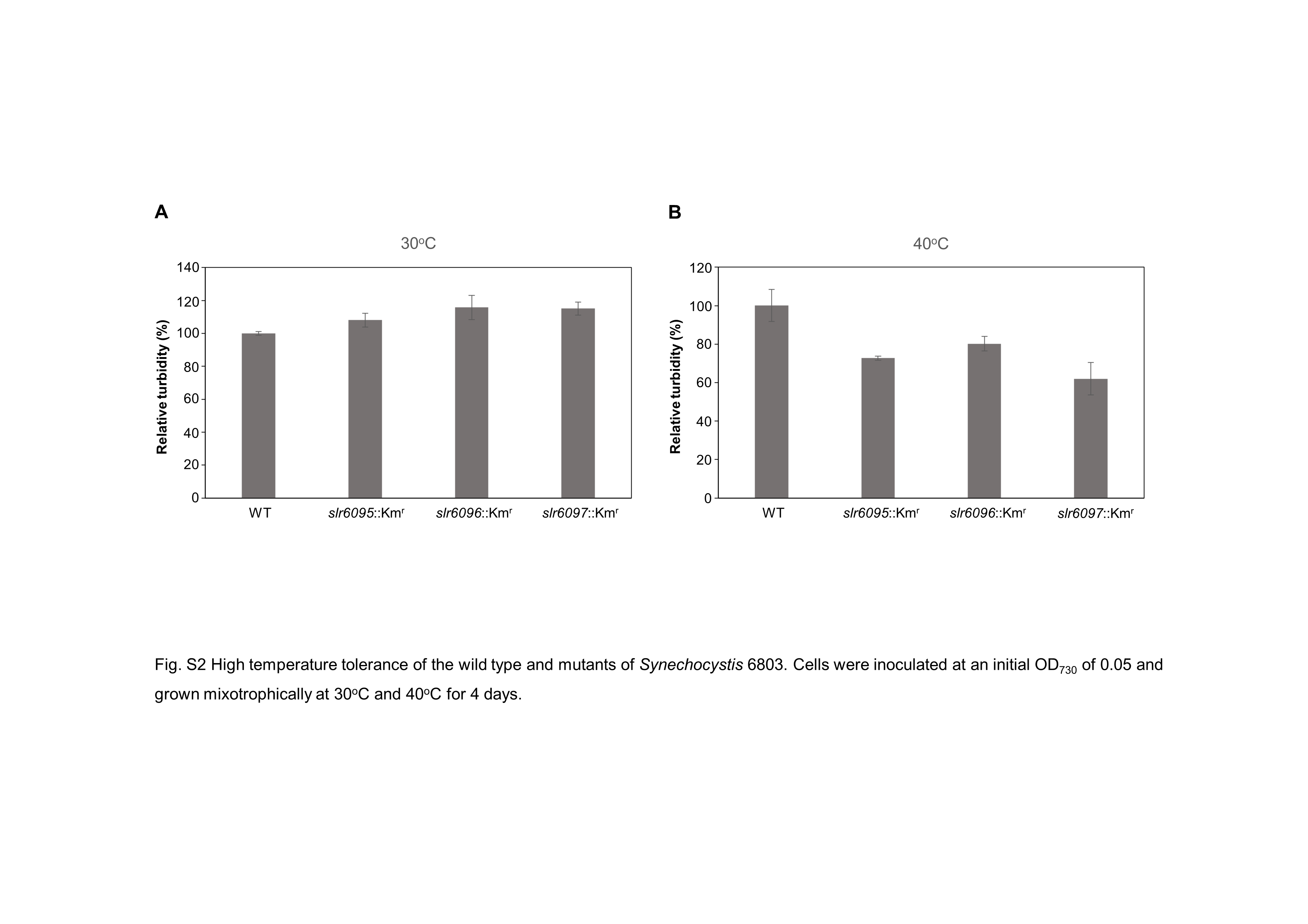

Supplement: Supplementary file 4 [file Image_2.TIF]
